# Increasing negative lymph node count predicts favorable OS and DSS in breast cancer with different lymph node-positive subgroups

**DOI:** 10.1371/journal.pone.0193784

**Published:** 2018-03-19

**Authors:** Xin Zhao, Jing Wei, Xiaoxin Li, Haochang Yang, Pei Wang, Susheng Cao

**Affiliations:** 1 Department of General Surgery, Tianjin First Central Hospital,Tianjin,China; 2 Department of Thoracic Surgery, Xuzhou Central Hospital, The Affiliated Xuzhou Hospital of Medical College of Southeast University, Xuzhou, China; 3 Department of Pathology, Xuzhou Central Hospital, The Affiliated Xuzhou Hospital of Medical College of Southeast University, Xuzhou, China; 4 College of Clinical Medicine, Binzhou Medical University, Yantai, China; 5 Department of Breast Surgery, Xuzhou Central Hospital, The Affiliated Xuzhou Hospital of Medical College of Southeast University,Xuzhou, China; University of North Carolina at Chapel Hill School of Medicine, UNITED STATES

## Abstract

Adequate lymph node evaluation is recommended for optimal staging in patients with malignant neoplasms including breast cancer. However, the role of negative lymph nodes (LNs) remains unclear in breast cancer according to N substage (N1, N2, and N3). In this study, for the first time, we analyzed the prognostic significance of negative LNs in breast cancer patients. A critical relationship was observed between negative LN count and survival, independent of patient characteristics and other related molecular variables including estrogen receptor (PR) status, progesterone receptor (ER) status, human epidermal growth factor receptor 2 (HER2) status, depth of tumor invasion and degree of differentiation. This research is of great importance in providing more information about the prognosis of breast cancer by statistical analysis of negative lymph nodes and can serve as a useful supplement to the current pathological system.

## Introduction

Breast cancer is one of the most common carcinomas worldwide, especially among females [1]. The prognosis of patients with early stage breast cancer is favorable, but patients with lymph node (LN) metastasis usually have a poor prognosis; the more metastatic lymph nodes identified, the worse the long-term survival [2]. In the American Joint Committee on Cancer (AJCC)/International Union Against Cancer (UICC) tumor-node-metastasis (TNM) classification [3], positive LNs are classified into 3 subgroups: N1 (1–3 LNs), N2 (4–9 LNs) and N3 (10 or more LNs). The staging system can discriminate prognostic groups very well. However, it is difficult to correctly assess a patient’s TNM classification. For example, one of the classic questions is the minimum number of LNs that should be dissected. Unfortunately, the number reported in the literature is inconsistent, ranging from 5 to 15 [4–8].

In addition to the total number of LNs excised, many other parameters have been developed and validated for various malignancies, such as number of negative lymph nodes [9,10], ratio of involved to removed nodes (lymph node ratio, LNR) [11,12], and log odds of positive lymph nodes (LODDS) [13,14], to aid the TNM system in predicting the outcome more accurately. However, few studies have focused on the relationship between the number of negative LNs and prognosis of breast cancer. Kuru [9] compared the roles of total number of nodes removed, negative nodes removed, and ratio of positive nodes to removed nodes in breast cancer using small samples from a single institute. Vinh-Hung et al [15] investigated the effect of number of negative nodes on survival in early breast cancer using the Surveillance Epidemiology and End Results (SEER) database. To the best of our knowledge, this study is the first to evaluate the role of negative LN count in breast cancer according to N substage (N1, N2, and N3) using a population-based database.

## Materials and methods

### Patients

Patients were collected from the SEER database (2004–2013), which is a population-based cancer registry in the United States. The National Cancer Institute’s SEER*Stat software (Version 8.2.0) was applied to identify patients with breast cancer. Included in the study were patients more than 18 years old, who had received surgical treatment and a pathologically confirmed diagnosis of breast cancer. The exclusion criteria were as follows: (1) patients for whom the number of examined or positive regional lymph nodes was 0 or unknown; (2) patients with distant metastasis (M1); (3) patients with a history of prior malignancy; and (4) patients with chemotherapy or radiotherapy before surgery. We retrieved baseline and clinical characteristics, including sex, age, race, tumor grade, T stage according to the 6th and 7th edition of AJCC criteria, estrogen receptor (ER) status, progesterone receptor (PR) status and human epidermal growth factor receptor 2 (HER2) status. All methods were carried out in accordance with relevant guidelines and regulations. All experimental protocols were approved by the Bioethics Committee of the Affiliated XuZhou Hospital of Medical College of Southeast University, China. Informed consent was obtained from all subjects in the SEER database.

### Statistical analysis

Data about baseline and clinical characteristics were presented as count and percent values. The selection of the subgroups in the negative number of lymph nodes was determined by X-tile software (Yale University, Version 3.6.1). Overall survival (OS) and disease-specific survival (DSS) were evaluated by the Kaplan-Meier method. OS was calculated from the time of initial diagnosis to last follow-up or death, and DSS was defined as the interval from diagnosis until death due to breast cancer. Multivariate analyses for OS and DSS of breast cancer were performed by Cox regression models with adjusted hazard ratios (HRs). *P* values < 0.05 were considered statistically significant. All analyses were conducted utilizing PASW Statistics 18 software.

## Results

A total of 125981 patients with breast cancer and positive LNs were selected from the SEER database, including 87523 cases with N1 substage, 26350 cases with N2 substage, and 12108 cases with N3 substage. Baseline and clinical characteristics are shown in **Table 1**. Most patients were female (99.1%), and the median age was 56 years old. The majority of patients had early T stage (T1 accounting for 37.7%, T2 accounting for 44.7%). The number of negative LNs for the cohort ranged from 0 to 77, and the median number was 8.

**Table 1:** Demographic and tumor characteristics for breast cancer patients with positive lymph nodes.

Characteristics	All patients (n = 125981)	N1 Substage (n = 87523)	N2 Substage (n = 26350)	N3 Substage (n = 12108)	P
**Age, years**					
Median	56	56	56	57	<0.001
**Sex**					
Female	124813 (99.1%)	86732 (99.1%)	26091 (99.0%)	11990 (99.0%)	0.425
Male	1168 (0.9%)	791 (0.9%)	259 (1.0%)	118 (1.0%)	
**Race**					
White	99520 (79.0%)	69426 (79.3%)	20537 (77.9%)	9557 (78.9%)	<0.001
Black	15258 (12.1%)	10151 (11.6%)	3531 (13.4%)	1576 (13.0%)	
Others	11203 (8.9%)	7946 (9.1%)	2282 (8.7%)	975 (8.1%)	
Grade*					
Well or moderately differentiated	67652 (53.7%)	50022 (57.2%)	12600 (47.8%)	5030 (41.5%)	<0.001
Poorly differentiated or undifferentiated	53470 (42.4%)	34228 (39.1%)	12719 (48.2%)	6523 (53.9%)	
T stage**					
T1	47554 (37.7%)	39430 (45.1%)	6190 (23.5%)	1934 (16.0%)	<0.001
T2	56336 (44.7%)	37847 (43.2%)	13055 (49.5%)	5434 (44.9%)	
T3	13385 (10.6%)	6329 (7.2%)	4224 (16.0%)	2832 (23.4%)	
T4	6831 (5.4%)	2702 (3.1%)	2447 (9.3%)	1682 (13.9%)	
**ER status**					
Positive	96859 (76.9%)	68583 (78.4%)	19679 (74.7%)	8597 (71.0%)	<0.001
Negative	25014 (19.9%)	16120 (18.4%)	5777 (21.9%)	3117 (25.7%)	
Others	4108 (3.2%)	2820 (3.2%)	894 (3.4%)	394 (3.3%)	
**PR status**					
Positive	82663 (65.6%)	59181 (67.6%)	16510 (62.7%)	6972 (57.6%)	<0.001
Negative	38120 (30.3%)	24789 (28.3%)	8707 (33.0%)	4624 (38.2%)	
Others	5198 (4.1%)	3553 (4.1%)	1133 (4.3%)	512 (4.2%)	
**HER2 status**					<0.001
Positive	8043(6.4%)	5473(6.3%)	1735(6.6%)	835(6.9%)	
Negative	38959(30.9%)	28146(32.2%)	7436(28.2%)	3377(27.9%)	
Others	78979(62.7%)	53904(61.6%)	17179(65.2%)	7896(65.2%)	
**NO. of negative LNs**					
0–3	33826 (26.9%)	22409 (25.6%)	5402 (20.5%)	6015 (49.7%)	<0.001
4–7	25315 (20.1%)	15520 (17.7%)	6724 (25.5%)	3071 (25.4%)	
8–11	25037 (19.9%)	17404 (19.9%)	6092 (23.1%)	1541 (12.7%)	
>11	41803 (33.2%)	32190 (36.8%)	8132 (30.9%)	1481 (12.2%)	

*Grade information missing for 4859 patients (3.9%).

**T stage information missing for 590 patients (0.5%).

ER = estrogen receptor, PR = progesterone receptor, HER2 = human epidermal growth factor receptor 2.

**Fig 1:**
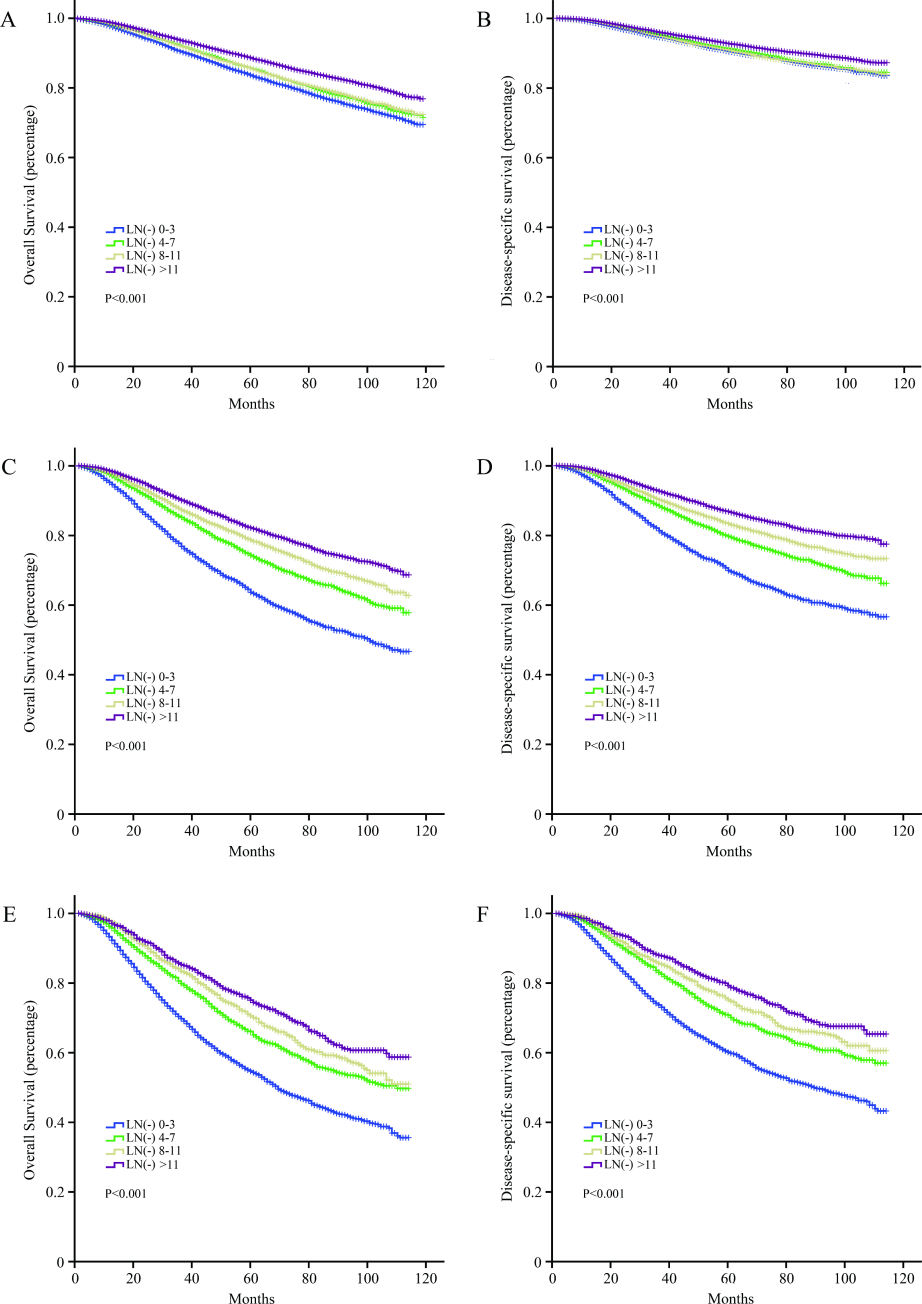
The OS of substage N1, N2 and N3 for breast cancer is shown in A, C and E, respectively. The DSS of substage N1, N2 and N3 for breast cancer is shown in B, D and F, respectively.

During the follow-up period, 21679 (17.2%) patients in the entire cohort died. Overall, 14729 patients died from breast cancer, accounting for 67.9% (14729/21679) of total deaths. Taking into consideration the optimal cutoff number of negative LNs acquired by X-tile software in N1, N2 and N3 substage and the number of negative LNs in each subgroup, four negative LNs were categorized as an interval. The N1, N2 and N3 substage were divided into 4 subgroups: 0–3, 4–7, 8–11 and more than 11. OS and DSS for N1, N2 and N3 substage stratified by 4 subgroups are presented in **Fig 1A, 1B, 1C, 1D, 1E and 1F**. For all three N substages, the differences in both OS and DSS were statistically significant (*P* < 0.001). It should be noted that there was no difference between N1 patients with 4–7 negative LNs and those with 8–11 for OS (*P* > 0.05). No significant differences were observed among N1 patients with subgroups 4–7, 8–11 and more than 11 for DSS (*P* > 0.05). All the survival curves indicated that patients with more negative LNs had better outcomes, especially for N2 and N3 substage.

The effect of negative LNs on prognosis was evaluated by multivariate analyses (**Tables 2 and 3**). The number of negative LNs was an independent prognostic factor for OS and CSS in each N substage. A larger number of negative LNs had a more positive effect on the prognosis of breast cancer. N2 disease was most influenced; patients with more than 11 negative LNs compared with patients with 0–3 negative LNs had 48.9% of the death risk for OS (95% CI, 0.455 to 0.526, respectively) and 43.9% for DSS (95% CI, 0.403 to 0.478, respectively). Additionally, T stage, ER status (positive vs. negative), PR status (positive vs. negative), HER2 status (positive vs. negative), grade (well or moderately differentiated vs. poorly differentiated or undifferentiated), age (≤55 years vs. >55 years) and race (white vs. black) were independent prognostic factors for all patients. Sex did not always influence mortality independently. For example, sex had no effect on either OS or DSS for N3 substage (*P* = 0.360, *P* = 0.495, respectively).

**Table 2:** Multivariate analyses for OS in breast cancer patients with positive lymph nodes.

Characteristics	N1 Substage (n = 87523)			N2 Substage (n = 26350)			N3 Substage (n = 12108)		
	HR	95% CI	P	HR	95% CI	P	HR	95% CI	P
**Age, years**									
≤55	1.0	Reference		1.0	Reference		1.0	Reference	
>55	2.414	2.315–2.518	<0.001	1.765	1.672–1.862	<0.001	1.493	1.401–1.592	<0.001
**Sex**									
Male	1.0	Reference		1.0	Reference		1.0	Reference	
Female	0.560	0.478–0.656	<0.001	0.693	0.549–0.876	0.002	0.860	0.629–1.177	0.347
**Race**									
White	1.0	Reference		1.0	Reference		1.0	Reference	
Black	1.348	1.279–1.422	<0.001	1.499	1.402–1.603	<0.001	1.481	1.362–1.611	<0.001
Others	0.755	0.697–0.818	<0.001	0.806	0.724–0.898	<0.001	0.752	0.660–0.856	<0.001
Grade*									
Well or moderately differentiated	1.0	Reference		1.0	Reference		1.0	Reference	
Poorly differentiated or undifferentiated	1.407	1.348–1.470	<0.001	1.408	1.329–1.491	<0.001	1.434	1.336–1.540	<0.001
T stage**									
T1	1.0	Reference		1.0	Reference		1.0	Reference	
T2	1.821	1.743–1.903	<0.001	1.524	1.415–1.641	<0.001	1.455	1.312–1.613	<0.001
T3	2.506	2.338–2.687	<0.001	2.060	1.886–2.250	<0.001	1.872	1.675–2.092	<0.001
T4	3.995	3.700–4.313	<0.001	2.606	2.379–2.856	<0.001	2.629	2.348–2.944	<0.001
**ER status**									
Positive	1.0	Reference		1.0	Reference		1.0	Reference	
Negative	1.422	1.338–1.511	<0.001	1.507	1.391–1.632	<0.001	1.412	1.289–1.548	<0.001
Others	1.265	1.060–1.510	0.009	1.308	1.018–1.680	0.035	1.502	1.132–1.992	0.005
**PR status**									
Positive	1.0	Reference		1.0	Reference		1.0	Reference	
Negative	1.338	1.265–1.415	<0.001	1.404	1.302–1.515	<0.001	1.460	1.337–1.593	<0.001
Others	1.183	1.007–1.390	0.041	1.161	0.924–1.459	0.200	1.209	0.932–1.567	0.153
**HER2 status**									
Positive	1.0	Reference	<0.001	1.0	Reference		1.0	Reference	
Negative	1.552	1.328–1.813	<0.001	1.342	1.110–1.622	0.002	1.636	1.313–2.038	<0.001
Others	1.620	1.398–1.878	<0.001	1.297	1.088–1.547	0.004	1.487	2.211–1.827	<0.001
**NO. of negative LNs**									
0–3	1.0	Reference		1.0	Reference		1.0	Reference	
4–7	0.823	0.776–0.871	<0.001	0.686	0.640–0.735	<0.001	0.741	0.686–0.801	<0.001
8–11	0.786	0.744–0.831	<0.001	0.589	0.547–0.634	<0.001	0.647	0.582–0.719	<0.001
>11	0.636	0.605–0.670	<0.001	0.489	0.455–0.526	<0.001	0.536	0.478–0.601	<0.001

*Grade information missing for 4859 patients (3.9%).

**T stage information missing for 590 patients (0.5%).

**Table 3:** Multivariate analyses for DSS in breast cancer patients with positive lymph nodes.

Characteristics	N1 Substage (n = 87523)			N2 Substage (n = 26350)			N3 Substage (n = 12108)		
	HR	95% CI	P	HR	95% CI	P	HR	95% CI	P
**Age, years**									
≤55	1.0	Reference		1.0	Reference		1.0	Reference	
>55	1.462	1.389–1.538	<0.001	1.297	1.220–1.378	<0.001	1.230	1.147–1.319	<0.001
**Sex**									
Male	1.0	Reference		1.0	Reference		1.0	Reference	
Female	0.688	0.535–0.885	0.004	0.889	0.643–1.231	0.479	0.875	0.606–1.265	0.477
**Race**									
White	1.0	Reference		1.0	Reference		1.0	Reference	
Black	1.332	1.246–1.423	<0.001	1.492	1.381–1.61	<0.001	1.431	1.304–1.570	<0.001
Others	0.757	0.684–0.838	<0.001	0.850	0.753–0.959	0.008	0.751	0.651–0.866	<0.001
Grade*									
Well or moderately differentiated	1.0	Reference		1.0	Reference		1.0	Reference	
Poorly differentiated or undifferentiated	1.874	1.767–1.988	<0.001	1.550	1.447–1.660	<0.001	1.515	1.398–1.642	<0.001
T stage**									
T1	1.0	Reference		1.0	Reference		1.0	Reference	
T2	2.144	2.017–2.280	<0.001	1.636	1.495–1.789	<0.001	1.430	1.275–1.603	<0.001
T3	3.386	3.105–3.692	<0.001	2.339	2.108–2.595	<0.001	1.908	1.687–2.158	<0.001
T4	5.418	4.927–5.958	<0.001	2.953	2.651–3.288	<0.001	2.632	2.322–2.982	<0.001
**ER status**									
Positive	1.0	Reference		1.0	Reference		1.0	Reference	
Negative	1.572	1.457–1.695	<0.001	1.662	1.517–1.822	<0.001	1.449	1.311–1.602	<0.001
Others	1.156	0.909–1.470	0.236	1.493	1.097–2.032	0.011	1.489	1.091–2.031	0.012
**PR status**									
Positive	1.0	Reference		1.0	Reference		1.0	Reference	
Negative	1.614	1.499–1.738	<0.001	1.523	1.392–1.665	<0.001	1.601	1.453–1.765	<0.001
Others	1.348	1.084–1.678	0.007	1.077	0.810–1.433	0.608	1.346	1.012–1.790	0.041
**HER2 status**									
Positive	1.0	Reference		1.0	Reference		1.0	Reference	
Negative	1.785	1.464–2.175	<0.001	1.470	1.185–1.824	<0.001	1.785	1.395–2.283	<0.001
Others	1.772	1.469–2.136	<0.001	1.313	1.074–1.604	0.008	1.591	1.263–2.003	<0.001
**NO. of negative LNs**									
0–3	1.0	Reference		1.0	Reference		1.0	Reference	
4–7	0.810	0.751–0.875	<0.001	0.661	0.610–0.715	<0.001	0.715	0.656–0.778	<0.001
8–11	0.799	0.743–0.859	<0.001	0.557	0.511–0.607	<0.001	0.616	0.548–0.693	<0.001
>11	0.630	0.589–0.673	<0.001	0.439	0.403–0.478	<0.001	0.511	0.450–0.581	<0.001

*Grade information missing for 4859 patients (3.9%).

**T stage information missing for 590 patients (0.5%).

## Discussion

The TNM classification system attempts to account for most basic parameters of cancer, to provide guidance for treatment planning and to predict the outcome. The number of positive LNs has been the focus on for a long time. In the present study, for the first time, we analyzed the prognostic significance of negative LNs in breast cancer patients. We observed a critical relationship between negative lymph node count and survival, independent of patient characteristics and other related molecular variables including PR status, ER status, HER2 ststus, depth of tumor invasion and degree of differentiation.

Although the number of negative LNs has an apparent effect on survival in each N stage, the mechanism underlying the relationship between negative LNs and survival remains unconfirmed. It has been reported that stage migration probably occurs due to under-evaluation of total lymph nodes, which has an intimate connection with the number of positive and negative LNs, thus influencing on pathological stage. Examining more lymph nodes can more accurately identify metastasis, thus avoiding misclassification of pathological stage [16].

Another possible hypothesis is that the number of negative LNs reflects the host lymphocytic reaction to tumor. The interaction between host and tumor contributes to the size or count of lymph nodes, and the increase of negative LNs indicates that the host takes a predominant position when competing with a tumor, and consequently obtains longer survival. A similar hypothesis has been associated with longer survival in colorectal cancer [17,18].

The number of lymph nodes informs the surgical approach, classification of pathological stage and institutional care. It has been reported that the number of lymph nodes may have a connection with better care overall and may not affect outcome directly [19–22]. There are also studies showing that the removal of more lymph nodes leads to a better prognosis [11]. Negative lymph nodes, clinically, imply that cancer cells were not observed using microscopy, instead of absolute no-tumor-metastasis, which means that there may be micro-metastasis of cancer cells. In other words, positive lymph nodes examined on routine pathological analysis do not necessarily reflect lymph node metastasis. For approximately 9%-30% of breast cancer patients, there are micro-metastases in their axillary lymph nodes that are undetectable by routine pathological examination [23]. Lymph node micro-metastasis is a strong risk factor for tumor-free survival and metastatic recurrence [24]. Therefore, the increase of the negative lymph node count, to a certain extent, can avoid the adverse effects of lymph node micro-metastasis and improve the prognosis of patients [25,26].

The rate of LNR was not used in this study to assess the prognosis of patients. LNR refers to the ratio of the number of metastatic lymph nodes to the total number of lymph nodes cleared. In recent years, many studies have indicated that LNR is superior to pN staging in reflecting axillary lymph node status and predicting prognosis [11, 27–31]. PN staging depends on the number of positive lymph nodes and the total number of lymph nodes cleared. Different from pN staging, LNR balances the potential influencing factors. It can better reflect axillary lymph node stage, as well as prognosis [32,33]. In a meta-analysis study, Woodward also suggested that LNR is better as a prognostic index than the number of metastatic lymph nodes [34].

In addition, we only selected patients from the SEER database to analyze survival and prognostic factors. It is necessary for us to obtain more detailed information to confirm the relationship between negative LNs and survival.

In conclusion, we confirmed, for the first time, the relationship between negative lymph node count and prognosis of breast cancer in substage N2 and N3. The analysis is consistent with the presentation of colorectal cancer: more negative LNs imply longer survival [35–38]. It is of great importance to provide more information about the prognosis of breast cancer by statistical analysis of negative lymph nodes, which can serve as a useful supplement to the current pathological system.

## Supporting information

S1 File Detailed data of all the patients from SEER. (XLSX)
